# Rubber band ligation versus haemorrhoidectomy for the treatment of grade II–III haemorrhoids: a systematic review and meta-analysis of randomised controlled trials

**DOI:** 10.1007/s10151-021-02430-x

**Published:** 2021-03-08

**Authors:** L. Dekker, I. J. M. Han-Geurts, H. D. Rørvik, S. van Dieren, W. A. Bemelman

**Affiliations:** 1 Department of Surgery, Amsterdam University Medical Center, Location AMC, Meibergdreef 9, 1105 AZ Amsterdam, The Netherlands; 2 Department of Surgery, Proctos Clinic, Bilthoven, The Netherlands; 3 Department of Surgery, Holbæk Hospital, Holbæk, Denmark; 4 Department of Acute and Digestive Surgery, Haukeland University Hospital, Bergen, Norway

**Keywords:** Haemorrhoids, Rubber band ligation, Haemorrhoidectomy, Complications, Reintervention

## Abstract

**Background:**

The aim of this study was to review clinical outcome of haemorrhoidectomy and rubber band ligation in grade II–III haemorrhoids.

**Methods:**

A systematic review was conducted. Medline, Embase, Cochrane Library, Clinicaltrials.gov, and the WHO International Trial Registry Platform were searched, from inception until May 2018, to identify randomised clinical trials comparing rubber band ligation with haemorrhoidectomy for grade II–III haemorrhoids. The primary outcome was control of symptoms. Secondary outcomes included postoperative pain, postoperative complications, anal continence, patient satisfaction, quality of life and healthcare costs were assessed. Preferred Reporting Items for Systematic Reviews and Meta-Analyses (PRISMA) guidelines were followed.

**Results:**

Three hundred and twenty-four studies were identified. Eight trials met the inclusion criteria. All trials were of moderate methodological quality. Outcome measures were diverse and not clearly defined. Control of symptoms was better following haemorrhoidectomy. Patients had less pain after rubber band ligation. There were more complications (bleeding, urinary retention, anal incontinence/stenosis) in the haemorrhoidectomy group. Patient satisfaction was equal in both groups. There were no data on quality of life and healthcare costs except that in one study patients resumed work more early after rubber band ligation.

**Conclusions:**

Haemorrhoidectomy seems to provide better symptom control but at the cost of more pain and complications. However, due to the poor quality of the studies analysed/it is not possible to determine which of the two procedures provides the best treatment for grade II–III haemorrhoids. Further studies focusing on clearly defined outcome measurements taking patients perspective and economic impact into consideration are required.

## Introduction

Haemorrhoids are one of the most common proctological disorders with an incidence of about 9/1000 patients per year in the Netherlands [1] and a prevalence up to 39% in the general population [2]. Treatment consists initially of conservative measures such as lifestyle advice, diet and toilet behaviour. In addition, there are various surgical options, Haemorrhoidectomy is considered the gold standard and this was recently confirmed in a British trial and systematic review [3, 4]. The most common minimally invasive procedure is rubber band ligation (RBL). Other minimally invasive procedures are sclerotherapy and laser treatment. These treatments are usually reserved for grade I and II haemorrhoids, although RBL is also used for grade III [5, 6]. Grade III and IV haemorrhoids can be treated with open haemorrhoidectomy, semi-closed haemorrhoidectomy, and stapled haemorrhoidectomy with possibly mucopexy or haemorrhoidal artery ligation (HAL).

Many studies and meta-analyses have been published on the subject of haemorrhoid treatment. All these studies focus on groups of comparable surgical procedures. It is common to distinguish between minimally invasive treatment for grade II and III diseases (sclerotherapy and RBL) and surgical procedures for grade III and IV haemorrhoids (haemorrhoidectomy and stapled haemorrhoidectomy). However, the criteria for selecting a minimally invasive treatment versus an operation are not always that evident. There is obviously an overlap in indication, as has become clear from several surveys amongst treating surgeons [7, 8]. There are few trials comparing the clinical outcome of the two most common treatments RBL and haemorrhoidectomy. A systematic review from 2005, updated in 2016**,** of 3 small heterogeneous trials concluded that RBL leads to a higher recurrence rate, but on the other hand less pain, fewer complications, and a less stressful experience for the patient [9, 10].

It remains unclear which of the two most common procedures is preferable as regards healthcare costs. There are hardly any studies investigating the cost effectiveness of the various treatments. Only 1 study compared costs of stapled haemorrhoidopexy with RBL in grade II haemorrhoids with results in favour of RBL [11]. A recent study from 2016 compared HAL with RBL, with HAL clearly entailing higher costs, even though the analysis includes the possibility of repeated RBL treatments [12]. Since haemorrhoidal disease is a benign condition, the main goal of treatment is the resolution of symptoms and improvement of patient wellbeing. It is, therefore, important to include patient-related outcomes when determining the best treatment.

The aim of this systematic review was to assess the literature on the clinical effectiveness (including patient-related outcomes) and cost effectiveness of RBL versus haemorrhoidectomy in patients with symptomatic grade II and III haemorrhoids.

## Materials and methods

This systematic review was undertaken in accordance with the Preferred Reporting Items for Systematic Reviews and Meta-Analyses (PRISMA) guidelines [13]. To reduce the risk of bias, a study protocol was made at an early stage and stated precise eligibility criteria. The protocol was registered in PROSPERO (registration number CRD42018102000) [14].

### Search strategy

A comprehensive literature search was carried out from inception until May 2018, using a combination of free-text terms and controlled vocabulary. Medline, Embase, Cochrane Library, Clinicaltrials.gov, and the WHO International Trial Registry Platform were searched to identify randomised clinical trials comparing RBL with haemorrhoidectomy. The references of the identified trials were also searched to find additional trials for inclusion. Only studies written in English were included. There were no restrictions on publication year or publication status.

### Search terms

The following search terms were used:

(“Hemorrhoids”[Mesh] OR hemorrhoid*[tiab] OR haemorrhoid*[tiab] OR piles[tiab]) AND (“Ligation”[Mesh] OR ligature*[tiab] OR ligation*[tiab] OR band*[tiab]) AND (“Surgical Procedures, Operative”[Mesh:NoExp] OR “Hemorrhoidectomy”[Mesh] OR “Diathermy”[Mesh] OR “Electrocoagulation”[Mesh] OR “Lasers”[Mesh] OR hemorroidectom*[tiab] OR haemorroidectom*[tiab] OR hemorrhoidectom*[tiab] OR haemorrhoidectom*[tiab] OR hemorrhoid excison*[tiab] OR haemorrhoid excison*[tiab] OR Milligan-Morgan[tiab] OR ferguson[tiab] OR ligasure[tiab] OR diathermy[tiab] OR harmonic scapel[tiab] OR electrocauter*[tiab] OR laser*[tiab] OR thermocoagulation[tiab]).

### Inclusion and exclusion criteria

Randomised controlled trials (RCTs) comparing RBL to/with haemorrhoidectomy in grade II–III haemorrhoids according to Goligher’s classification were included in this systematic review. Only studies considering non-emergency procedures in adult patients and reporting of the required outcomes were included. Adult patients (18 years or older) were included and all techniques (open, semi-closed, and closed) or instruments (scissors, knife, diathermy, LigaSure, and harmonic scalpel) used for haemorrhoid excision were included. Non-randomised studies and studies not in English language were excluded.

### Quality assessment

The methodological quality of the included studies was assessed using the following Cochrane Risk of Bias assessment tool: sequence generation, allocation concealment, blinding of participants and personnel, blinding of outcome assessors, incomplete outcome data, selective outcome reporting and other sources of bias [15]. Grading of Recommendations Assessment, Development, and Evaluation (GRADE) [16] was used to assess the quality (certainty) of evidence. It grades evidence as high, moderate, low or very low quality. Judgements included risk of bias, inconsistency, indirectness, imprecision and other considerations.

### Outcomes of interest

The primary outcome was control of haemorrhoidal disease defined by need for retreatment within 1 year or by self-reported residual complaints. The secondary outcomes were postoperative pain, postoperative complications (bleeding requiring admission and/or reoperation, sepsis, anal stenosis, anal incontinence), anal continence (if measured by a validated patient-reported outcome measure), patient satisfaction, quality of life (if measured by a validated patient-reported outcome measure), and health-costs. All complications reported (by studies) were added and reported individually.

### Data collection

Literature search results were uploaded to Covidence Software. This is a Cochrane-supported software program that can import citations, screen titles, abstracts and full text. Data selection and extraction was conducted in accordance with Population, Interventions, Comparison, Outcome (PICOs). Identified trials were screened by two independent investigators. Titles, abstracts and full text were screened by both reviewers against inclusion and exclusion criteria. Trials that were excluded were documented with reasons for exclusion recorded. Efforts were made to contact trial investigators to resolve questions about eligibility or missing data but did not lead to additional data. The reviewers were not blinded to the journal titles or to study authors or institutions.

### Statistical analysis

Binary data indicating number of patients with an event were analysed using a binomial model calculating risk ratio (RR) and 95% confidence interval (CI). The estimates from individual RCTs were pooled using the random-effects model. Statistical heterogeneity was explored by *χ*^2^ test and expressed as *I*^2^ and *p* value (considered significant if *p* < 0.05). The potential effect of predictors on the outcomes was investigated using a random-effects meta-regression model. Analyses were made using RevMan 5.3.5 (The Cochrane Collaboration) and RStudio.

## Results

**Fig. 1:**
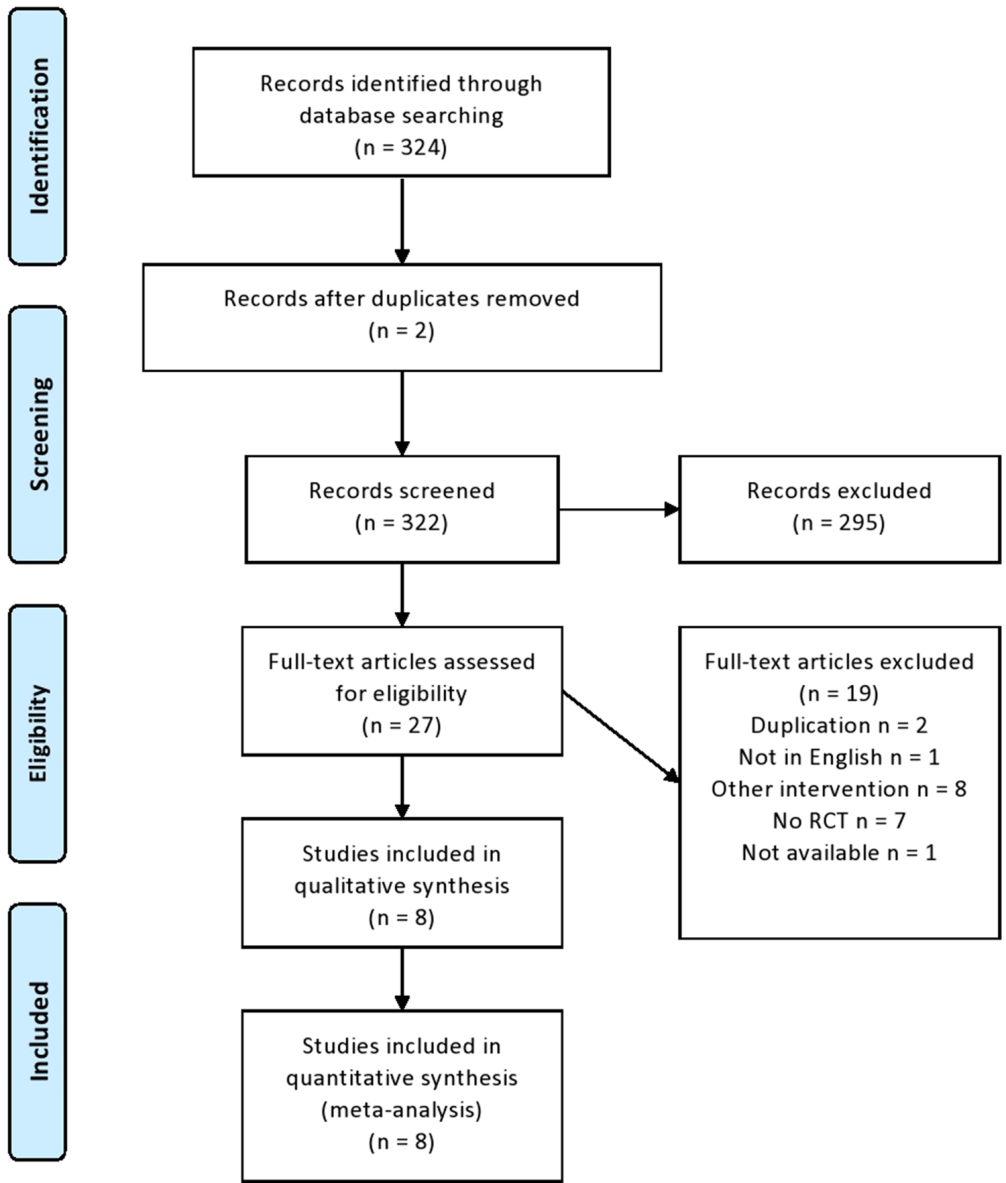
PRISMA flowchart of literature search

**Fig. 2:**
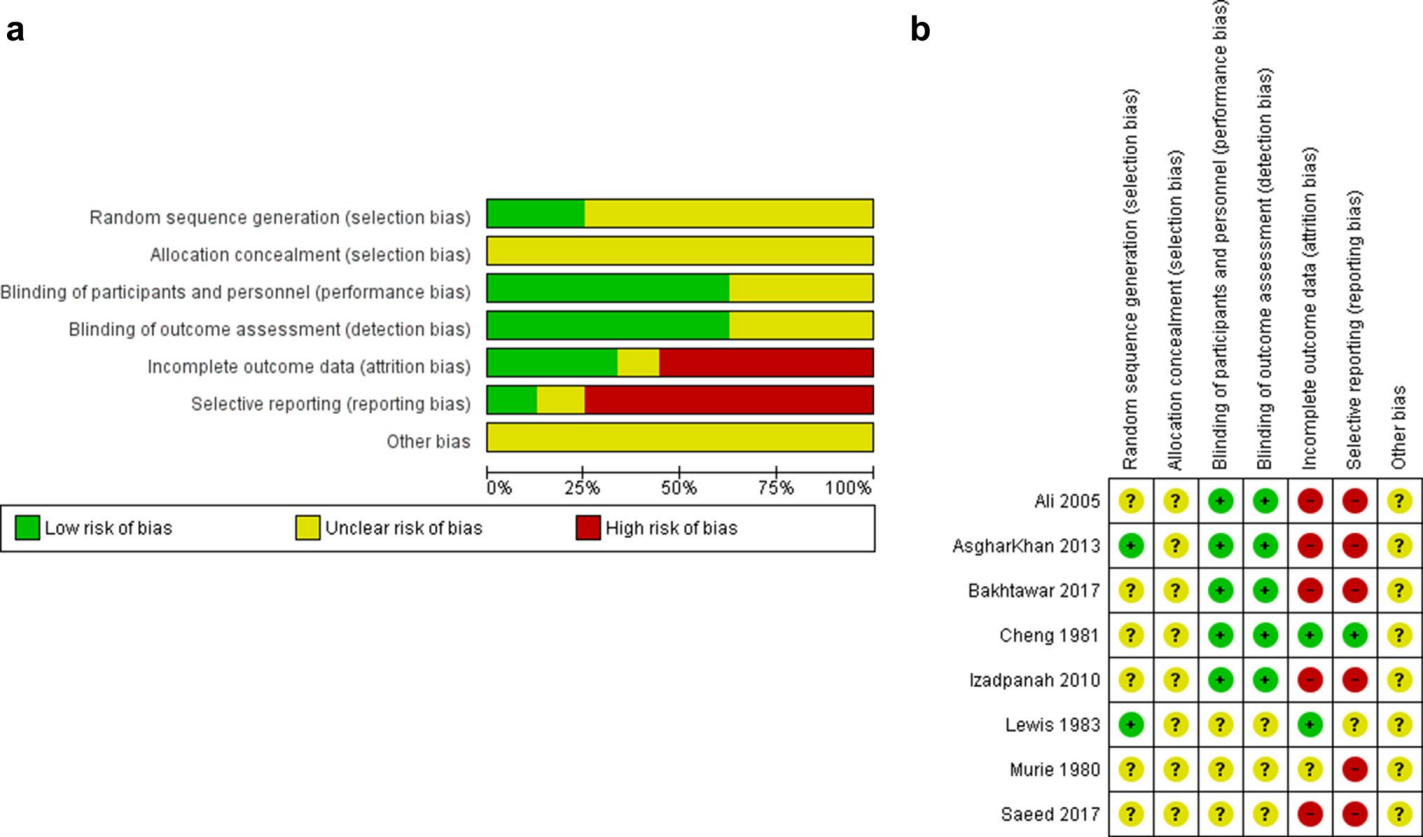
**a** Summary of risk of bias across included studies. **b** Summary of risk of bias for each included study

A total of 324 references were identified from the relevant electronic searches. Two duplicates were removed. Two hundred and ninety-five studies were excluded after screening titles and abstracts. Twenty-seven full-text studies were assessed for eligibility. Of these, 19 were excluded after full-text review. Eight RCTs were identified and included in the analyses (Fig. 1) [17–24]. The risk of bias in the included trials is summarised in Fig. 2a, b. The overall methodological quality of these studies was determined to be moderate. The eight trials contained a total of 1208 patients with second- and third-degree haemorrhoids, who underwent RBL or haemorrhoidectomy (608 versus 600, respectively). The characteristics of the studies are shown in Table 1.

**Table 1:** Characteristics of studies included in the meta-analysis

	Year	Country	Duration	II/III degree ratio	Total nr of patients	M/F ratio	Mean age (years)	Arms	Intervention	RBL	Haem	Technique RBL	Technique haem	Symptoms		Mean follow-up	Lost to follow-up
														Bleeding	Soiling		
Ali	2005	Pakistan	7 months	20/80	100	1.3:1	50	2	RBL vs. haem	50	50	2 bands per pile, 1 session	Milligan-Morgan	90	42	Till discharge	N/A
Asghar Khan	2013	Pakistan	18 months	55/65	120	1:0.2	39	2	RBL vs. haem	60	60	2 bands per pile	Milligan-Morgan	N/A	N/A	6 months	N/A
Bakhtawar	2017	Pakistan	5 months	255/279	534	1:0.2	43	2	RBL vs. haem	267	267	2 bands per pile	Milligan-Morgan	455	66	N/A	N/A
Cheng	1981	China	14 months	120/0	120	1:0.9	42	4	RBL vs. haem vs. sclerotherapy vs. anal dilatation	30	30	2 bands per pile	Milligan-Morgan	82	N/A	N/A	N/A
Izadpanah	2010	Iran	20 months	72/78	150	1:1.5	40	3	RBL vs. haem vs electrotherapy	51	47	N/A	Ferguson	120	N/A	3 months	N/A
Lewis	1983	England	35 months	23/33	56	1:0.8	48	3	RBL vs. haem vs. cryotherapy vs. anal dilatation	30	26	Max 3 bands, max 3 sessions	Milligan-Morgan	N/A	N/A	N/A	4
Murie	1980	Scotland	24 months	32/56	88	1:0.5	52	2	RBL vs. haem	43	45	2 bands per pile	Milligan-Morgan	84	–	12 months	4
Saeed	2017	Pakistan	39 months	60/80	140	1:0.2	41	2	RBL vs. haem	70	70	Max 2 bands per pile, 1 session	Milligan-Morgan	115	16	N/A	N/A

*haem* haemorrhoidectomy

### Recurrence and need for retreatment

**Fig. 3:**
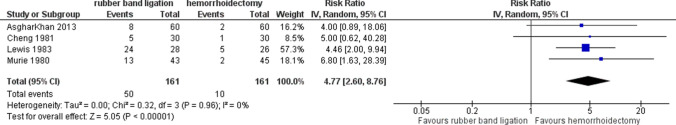
Recurrence rate. Relative risk values are shown with 95% confidence intervals

Recurrence was identified as outcome in 4 of the 8 trials. RBL led to more recurrence than haemorrhoidectomy (4 studies, 322 patients, random effects; RR 4.77 (95% CI 2.60–8.76); *p* < 0.001) as shown in Fig. 3). The index of heterogeneity between studies was assessed (*I*^2^) for a fixed effects model, and was low (0%). Recurrence of disease was established in different ways: need of reintervention [22]; diminishment of bleeding and prolapse [23] and recurrence of complaints [18, 20]. GRADE evidence for recurrence within all included studies was very low (Table 2).

**Table 2:** GRADE evidence profile; quality assessment per outcome

Certainty assessment							№ of patients		Effect		Certainty	Importance
№ of studies	Study design	Risk of bias	Inconsistency	Indirectness	Imprecision	Other considerations	Rubber band ligation	Hemorrhoidectomy	Relative (95% CI)	Absolute (95% CI)		
Urinary retention												
6	Randomised trials	Serious^a,b^	Not serious	Serious^c^	Not serious	None	15/527 (2.8%)	107/527 (20.3%)	**RR 0.15** (0.09 to 0.25)	**173 fewer per 1.000** (from 185 to 152 fewer)	⨁⨁◯◯ LOW	Critical
Postoperative pain												
7	Randomised trials	Serious^a,b,d,e^	Not serious	Serious^c^	Not serious	None	68/557 (12.2%)	438/553 (79.2%)	**RR 0.17** (0.11 to 0.28)	**657 fewer per 1.000** (from 705 to 570 fewer)	⨁⨁◯◯ LOW	Critical
Postoperative bleeding												
7	Randomised trials	Serious^a,b,d^	Not serious	Serious^c^	Serious^f^	None	22/557 (3.9%)	84/553 (15.2%)	**RR 0.31** (0.15 to 0.66)	**105 fewer per 1.000** (from 129 to 52 fewer)	⨁◯◯◯ VERY LOW	Critical
Anal stenosis												
5	Randomised trials	Serious^a,b,d^	Not serious	Serious^c^	Not serious	None	1/470 (0.2%)	26/472 (5.5%)	**RR 0.11** (0.03 to 0.38)	**49 fewer per 1.000** (from 53 to 34 fewer)	⨁⨁◯◯ LOW	Critical
Recurrence												
4	Randomised trials	Serious^a,b,d,g^	Serious^h^	Serious^c^	Serious^f^	None	50/161 (31.1%)	10/161 (6.2%)	**RR 4.77** (2.60 to 8.76)	**234 more per 1.000** (from 99 to 482 more)	⨁◯◯◯ VERY LOW	Critical
Anal incontinence												
3	Randomised trials	Serious ^a,b,d^	Not serious	Serious^c^	Serious^f^	None	0/120 (0.0%)	5/116 (4.3%)	**RR 0.16** (0.02 to 1.28)	**36 fewer per 1.000** (from 42 fewer to 12 more)	⨁◯◯◯ VERY LOW	Critical

*CI* Confidence interval; *MD* Mean difference; *RR* Risk ratio

^a^Lack of allocation concealment and lack of blinding in all studies. This, however, is unavoidable in most surgical RCTs

^b^Incomplete accounting of patients and outcome events. Of all studies, only three mentioned the loss to follow-up

^c^Saeed, Murie, Bakhtawar, Khan, Ali not mentioned prespecified primary and secondary outcomes

^d^No information on how the randomisation sequence was generated

^e^Unclear how postoperative pain was scored

^f^Several outcomes were reported in few studies and few patients and few events

^g^Unclear how patient satisfaction was defined

^h^Unclear how recurrence was defined

### Postoperative pain

**Fig. 4:**
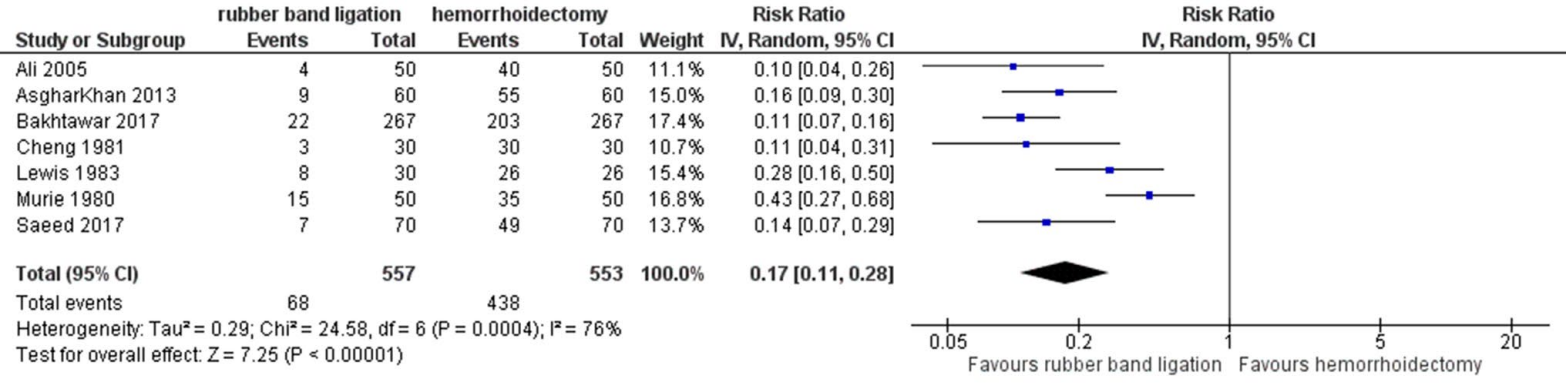
Postoperative pain. Relative risk values are shown with 95% confidence intervals

Patients experienced less post-procedural pain after RBL as demonstrated in Fig. 4 (7 studies, 1110 patients, RR 0.17 (95% CI 0.11–0.28); *p* < 0.001). Heterogeneity between studies was moderate (*I*^2^ = 76%, *p* < 0.001). This statistical heterogeneity between the studies may be explained by variations in the method used to measure the postoperative pain or the moment it was scored. Often it was not even mentioned [17, 19, 20, 24]. Only Izadpanah et al. used the visual analog scale to measure the pain score which was in favor of RBL (5 versus 8) [21]. The GRADE-rated evaluation showed low quality of evidence due to downgrading on risk of bias, indirectness and imprecision.

### Postoperative bleeding

**Fig. 5:**
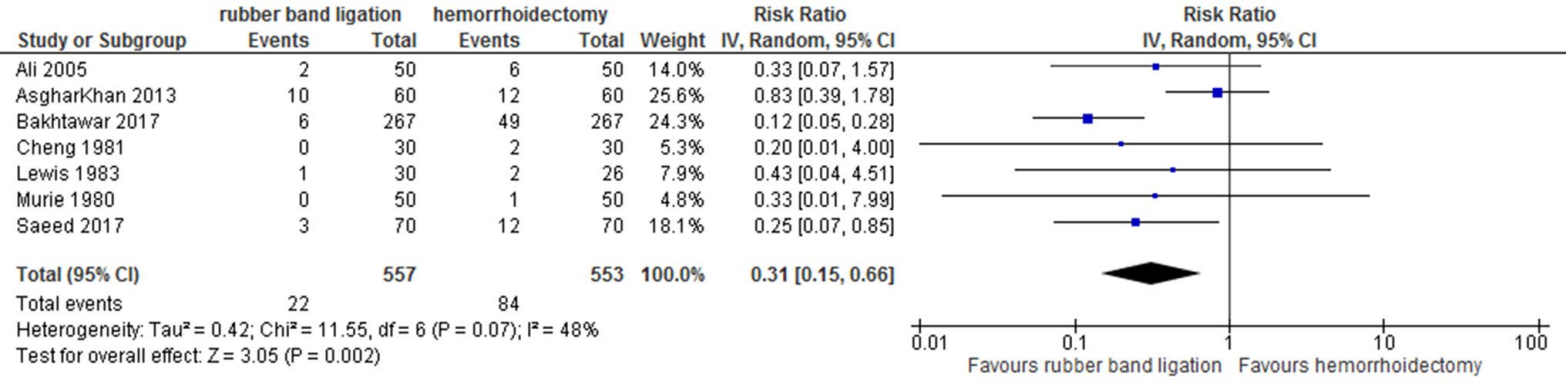
Postoperative bleeding. Relative risk values are shown with 95% confidence intervals

Seven studies including 1110 patients and 84 events described postoperative bleeding as an outcome. This was less common following RBL [random effects; RR 0.31 (95% CI 0.15–0.66); *p* = 0.002]. Heterogeneity between studies was moderate (*I*^2^ = 48%) (Fig. 5). None of the included studies describes how this outcome was defined. Following haemorrhoidectomy, bleeding required reintervention in 15 patients [17, 18, 20, 22–24], Only Murie et al. reported that transfusion was the intervention used for their only patient with bleeding after haemorrhoidectomy. In the RBL arm, one patient needed readmission, no reintervention was described [22]. Quality of evidence was graded as very low for postoperative bleeding due to downgrading on risk of bias, indirectness and imprecision.

### Urinary retention

**Fig. 6:**
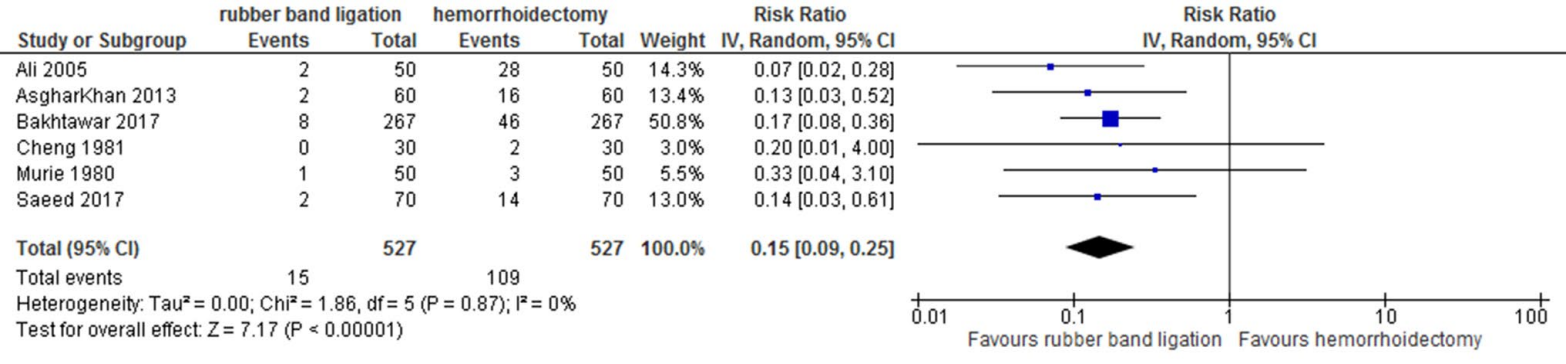
Urinary retention. Relative risk values are shown with 95% confidence intervals

Six studies reported data on urinary retention. All of them concluded that urinary retention requiring a urinary catheter is more common after haemorrhoidectomy than after RBL (6 studies, 1054 patients, random effects; RR 0.15 [95% CI 0.09–0.25]; *p* < 0.001) (Fig. 6). The rate of urinary retention was 0–4% after RBL versus 6.7–56% after haemorrhoidectomy. Due to downgrading on risk of bias and indirectness quality of evidence was assessed low.

### Anal continence and anal stenosis

**Fig. 7:**
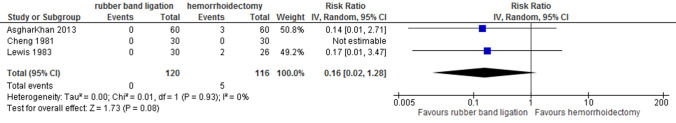
Anal incontinence. Relative risk values are shown with 95% confidence intervals

Anal incontinence was scored in 3 studies [17, 19, 21] and none of them found incontinence after RBL [236 patients, random effects; RR 0.16 [95% CI 0.02–1.28] *p* = 0.080) (Fig. 7)]. Ashghar et al. described incontinence in the haemorrhoidectomy group in, respectively, 5% and 7.7% of patients [19]. GRADE evidence for anal incontinence in all 3 studies was very low due to downgrading on risk of bias, indirectness and imprecision.

**Fig. 8:**
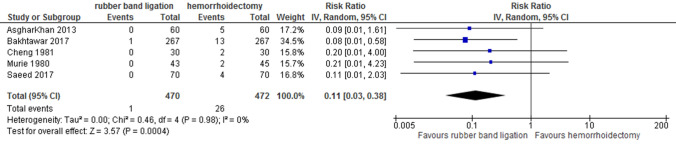
Anal stenosis. Relative risk values are shown with 95% confidence intervals

Five studies reported on anal stenosis (total of 942 patients, random effects; RR 0.11 [95% CI 0.03–0.38] *p* < 0.001) (Fig. 8). After haemorrhoidectomy, 1–8.3% of patients developed anal stenosis. Stenosis following RBL only occurred in one patient [19]. Quality of evidence was stated to be low for this outcome.

### Patient satisfaction

Murie et al. performed a patient assessment in which 93% of patients undergoing haemorrhoidectomy had an excellent to moderately successful result versus 88% of patients undergoing rubber band ligation [22]. Ashgar et al. reported a patient satisfaction rate of 93% in the haemorrhoidectomy arm compared to 86% in the RBL arm [19]. This was due to the necessity of a repeat procedure in the RBL group. Regarding patient load, Saeed et al. reported a hospital stay of 2.5 days after haemorrhoidectomy versus 1 day after RBL [23]. Loss of working days following treatment was reported by Murie et al. favouring RBL (32 versus 3 days): this difference was statistically significant.

### Predictors for postoperative pain after RBL and haemorrhoidectomy

The variable significantly associated with more post-procedural pain was age, which explained part of the heterogeneity. A meta-regression showed an age corrected RR of 0.23 for RBL compared to haemorrhoidectomy (95% CI 0.13–0.43, *p* < 0.001). Only 5 trials were analysed, as Cheng et al. did not mention the standard deviation [17]. A meta-regression on sex was not associated with postoperative pain and did not explain the heterogeneity (*p* = 0.560).

## Discussion

The present study gives an update of the results of the two most commonly used strategies in treatment of grade II and III haemorrhoids. The results of this review suggest that haemorrhoidectomy is superior to RBL in reducing symptoms but is associated with more postoperative pain and adverse events. The review included only RCTs. Studies otherwise designed would result in an increase of bias. The overall quality of the included studies based on the Cochrane Collaboration’s risk of bias tool was questionable. Incomplete outcome data (attrition bias) and selective reporting (reporting bias) were the major drawbacks. Furthermore, an important limitation was the lack of or poor definition of outcome measurements. The overall methodological quality of the included studies is moderate. Unfortunately, none of the included studies described the randomisation process and three of the eight studies compared more procedures than the two we were interested in. The included studies did not all use the same techniques of haemorrhoidectomy and RBL applications and only 3 studies reported the length of follow-up which was, respectively, 3, 6 and 12 months [17, 21, 23]. The overall certainty of the evidence using the GRADE system was, therefore, low to very low (Table2). It should be noted that three studies [18, 22, 23] are not of recent date but we still consider these relevant since the surgical procedures discussed have not changed since.

We defined control of haemorrhoidal disease by need for retreatment within 1 year or by self-reported residual complaints. Four studies report on effect of treatment and/or recurrence but a definition or follow-up is not given making results hard to interpret. Three studies only mention effect of treatment on bleeding, prolapse [18, 23] or pruritus [22] while other symptoms of haemorrhoidal disease are not mentioned. This makes it difficult to comment on efficacy of treatment. Other trials reporting on the outcomes of these procedures also demonstrate a lower recurrence rate after haemorrhoidectomy with the same limitations [4, 9, 25]. Besides, should repeated banding be considered as recurrence or part of the treatment? For re-banding, for instance, two or three sessions are common and patients may find this a more agreeable than one operation if the results are comparable in the long run. Except for 2 trials [17, 24], which reported performing 1 session of RBL, none of the included trials describes the exact number of RBL sessions.

Overall, postoperative complications were more common after haemorrhoidectomy. Postoperative bleeding and pain were mentioned in all studies and was more common following haemorrhoidectomy. However, none of the studies defined bleeding and only 1 used a visual analog scale to assess postoperative pain [21]. In addition, the timing of these outcome assessments was not specified in most studies. Pain after RBL has been analysed in other studies comparing RBL with more invasive procedures and was found to be less severe after RBL [12]. In a study by Watson et al. [26], 183 patients were asked to rate their pain on a scale of 1–5 at different time points after RBL. The most severe pain was experienced at 4 h following RBL and after 1 week, 75% of the patients did not experience any pain at all. In the HubBle trial, pain was less after RBL compared to a surgical procedure (HAL) at 1 day (3.4 versus 4.6) and 1 week (1.6 versus 3.1) following the procedure [12]. After 3 and 6 weeks, pain scores were similar in both groups.

Urinary retention occurred far more often after haemorrhoidectomy. Rates of urinary retention are reported in the literature: 2–34% after haemorrhoidectomy and 0–0.4% after RBL [3, 27, 28]. The mechanism responsible for urinary retention is thought to be the triggering of a reflex leading to inhibition of the detrusor muscle. Pain and stretching of the anal canal may induce this reflex. The extent of surgical resection is related to the risk of developing urinary retention, probably due to more postoperative oedema and pain [29].

Anal incontinence following haemorrhoidectomy was reported in 3 studies [18, 20, 22] ranging from 0 to 7.7%. Anal incontinence after RBL was not reported. This is in concordance with the recent literature [30]. However, none of the studies used a validated scoring system for anal incontinence. Other literature using the Vaizey or Cleveland incontinence score mention similar scores for RBL and HAL [12]. Anal stenosis was found in 1 patient after RBL and was not common after haemorrhoidectomy either (26/472) but this difference was significant. This stresses the importance of a careful surgical technique in performing haemorroidectomy which is sometimes is considered simple surgery.

Treatment patients complaining of haemorrhoids aims to improve these symptoms, making quality of life an essential marker of success. Patient satisfaction was similar between the groups but no validated questionnaires were used [20, 23, 24]. The literature on patient satisfaction following haemorrhoidal treatment is scarce. Brown et al. found in a study comparing RBL with HAL found that patient satisfaction after RBL did not differ from HAL in the long term [31].

Murie et al. reported 32 lost days of work after haemorrhoidectomy compared to 3 days after RBL [22]. Time until return to work and normal activities after haemorrhoidectomy has been reported to vary between 9 and 54 days [32]. This wide range can be due to the number of (one-, two-, three-) piles operated or the policy regarding postoperative pain management.

There are numerous studies on treatment of haemorrhoids with various techniques. This illustrates a lack of consensus about when to apply which technique for which symptoms. Treatment for a benign disease like haemorrhoids has to be safe and should be aimed at relieving symptoms. More conservative methods like RBL are reserved for grade II (but also III) haemorrhoids and more invasive surgical methods for grade III (but also II). That leaves a grey area in which the choice of treatment is not so evident. The gold standard for conservative methods is RBL and the gold standard for surgical procedures is haemorrhoidectomy [33]. Studies comparing these two methods are scarce and only 1 systematic review comparing 3 trials on this subject has been published [9].

Reliable outcome measurements relate to the definition of haemorrhoids. The choice of treatment is mostly based on gradation of haemorrhoids usually based on Goligher’s classification [34]. However, symptoms do not reliably relate to Goligher’s classification [35]. Clinical evaluation using only the Goligher scale could cause confusion regarding true symptomatic recurrence or symptom persistence. A more solid definition of failure or recurrence together with a validated score of symptoms is indispensable in evaluating treatment [36].

Van Tol et al. recently analysed outcome measurements used in trials on haemorrhoids [37]. Fifty-nine largely varying outcomes were identified. Based on these, the authors developed four different core areas: symptoms, complications, recurrence and resource use/economical impact. When we consider, these core areas in the analysed trials symptoms are only rarely described. None of the studies used a validated symptom score. Recurrence was reported in four studies and was more common following RBL. Complications (postoperative pain, anal stenosis/incontinence, bleeding and urinary retention) were mentioned in 6 studies. Resource use/economical impact was not addressed in any of the studies.

It is also important to realise that haemorrhoidal disease is currently one of the most common disabilities. The condition often leads to disruption in an individual’s personal and working life. Management has considerable cost implications, and therefore, economic consequences. None of the included trials mentions costs. Future studies should focus not only on and patient satisfaction with treatment but also on the economic impact of treatment.

## Conclusions

The results of this review suggest that haemorrhoidectomy offers better symptom control compared with rubber band ligation in patients with grade II–III haemorrhoids, but is accompanied by more postoperative pain and complications. The main conclusion, however, must be that the studies analysed are of poor quality, and therefore, no advice about treatment protocol can be given. Good quality trials with an emphasis on economic and patient-related outcomes are needed. A multicentre randomised trial comparing RBL with haemorrhoidectomy has recently been initiated in the Netherlands.

## References

[1] NIVEL, ‘Incidence and prevalence rates of Haemorrhoids in Dutch general practice classified by sex in 2017 (per 1000 patient years)’. Retrieved October 2020 [Online]. Available: https://www.nivel.nl/nl/nivel-zorgregistraties-eerste-lijn/jaarcijfers-aandoeningen-huisartsenregistraties.

[2] Riss S (2012). The prevalence of hemorrhoids in adults. Int J Colorectal Dis.

[3] Watson AJM (2016). Comparison of stapled haemorrhoidopexy with traditional excisional surgery for haemorrhoidal disease (eTHoS): a pragmatic, multicentre, randomised controlled trial. Lancet.

[4] Simillis C, Thoukididou SN, Slesser AAP, Rasheed S, Tan E, Tekkis PP (2015). Systematic review and network meta-analysis comparing clinical outcomes and effectiveness of surgical treatments for haemorrhoids. Br J Surg.

[5] Davis BR, Lee-Kong SA, Migaly J, Feingold DL, Steele SR (2018). The American Society of colon and rectal surgeons clinical practice guidelines for the management of hemorrhoids. Dis Colon Rectum.

[6] van Tol N, Kleijnen RR, Watson J, Jongen AJM, Altomare J, Qvist DF, Higuero SO, Muris T, Breukink J (2019). ‘European Society of ColoProctology (ESCP) Guideline for Haemorrhoidal Disease’, What do we know so far?. CardioVas Interv Radiol.

[7] van Tol RR, Bruijnen MPA, Melenhorst J, van Kuijk SMJ, Stassen LPS, Breukink SO (2018). A national evaluation of the management practices of hemorrhoidal disease in the Netherlands. Int J Colorectal Dis.

[8] Altomare DF (2018). Surgical management of haemorrhoids: an Italian survey of over 32 000 patients over 17 years. Color Dis.

[9] Shanmugam V, Thaha MA, Rabindranath KS, Campbell KL, Steele RJC, Loudon MA (2005). Systematic review of randomized trials comparing rubber band ligation with excisional haemorrhoidectomy. Br J Surg.

[10] Brown SR, Watson A (2016). Comments to “Rubber band ligation versus excisional haemorrhoidectomy for haemorrhoids”. Tech Coloproctol.

[11] McKenzie L (2010). ‘Economic evaluation of the treatment of grade II haemorrhoids: a comparison of stapled haemorrhoidopexy and rubber band ligation. Colorectal Dis.

[12] Brown SR (2016). Haemorrhoidal artery ligation versus rubber band ligation for the management of symptomatic second-degree and third-degree haemorrhoids (HubBLe): a multicentre, open-label, randomised controlled trial. Lancet.

[13] Moher D (2016). Preferred reporting items for systematic review and meta-analysis protocols (PRISMA-P) 2015 statement. Rev Esp Nutr Hum Diet.

[14] ‘National Institute for Health Research.’ [Online]. Available: https://www.crd.york.ac.uk/prospero/

[15] Higgins JPT (2011). The cochrane collaboration’s tool for assessing risk of bias in randomised trials’. BMJ.

[16] Guyatt GH (2008). GRADE: An emerging consensus on rating quality of evidence and strength of recommendations. BMJ.

[17] Ali U, Khan AS (2005). Rubber band ligation versus open haemorrhoidectomy: a study of 100 cases. J Postgrad Med Inst.

[18] Cheng FCY, Shum DWP, Ong GB (1981). The treatment of second degree haemorrhoids by injection, rubber band ligation, maximal anal dilatation, and haemorrhoidectomy: a prospective clinical trial. Aust N Z J Surg.

[19] Bakhtawar A, Khalid MA, Arshad A (2017). Comparison of Milligan - Morgan haemorrhoidectomy vs rubber band ligation in management of haemorrhoids. Med Forum Mon.

[20] Asghar Khan M (2013). Short term outcome of rubber band ligation versus open hemorrhoidectomy in terms of postoperative complications. J Med Sci.

[21] Izadpanah A, Hosseini S, Mahjoob M (2010). Comparison of electrotherapy, rubber band ligation and hemorrhoidectomy in the treatment of hemorrhoids: a clinical and manometric study. Middle East J Dig Dis.

[22] Lewis AAM, Rogers HS, Leighton M (1983). Trial of maximal anal dilatation, cryotherapy and elastic band ligation as alternatives to haemorrhoidectomy in the treatment of large prolapsing haemorroids. Br J Surg.

[23] Murie JA, MacKenzie I, Sim AJW (1980). Comparison of rubber band ligation and haemorrhoidectomy for second- and third-degree haemorrhoids: a prospective clinical trial. Br J Surg.

[24] Saeed MT, Ali Z, Khan SA (2017). Milligan - Morgan (Open) haemorrhoidectomy vs rubber band ligation. Pakistan J Med Heal Sci.

[25] Cocorullo G (2017). The non-surgical management for hemorrhoidal disease. A systematic review. G di Chir.

[26] Watson NFS, Liptrott S, Maxwell-Armstrong CA (2006). A prospective audit of early pain and patient satisfaction following out-patient band ligation of haemorrhoids. Ann R Coll Surg Engl.

[27] Zaheer S, Reilly WT, Pemberton JH, Ilstrup D (1998). Urinary retention after operations for benign anorectal diseases. Dis Colon Rectum.

[28] Toyonaga T (2006). Postoperative urinary retention after surgery for benign anorectal disease: potential risk factors and strategy for prevention. Int J Colorectal Dis.

[29] Kowalik U, Plante MK (2016). Urinary retention in surgical patients. Surg Clin North Am.

[30] Trenti L, Biondo S, Galvez A, Bravo A, Cabrera J, Kreisler E (2017). Distal Doppler-guided transanal hemorrhoidal dearterialization with mucopexy versus conventional hemorrhoidectomy for grade III and IV hemorrhoids: postoperative morbidity and long-term outcomes. Tech Coloproctol.

[31] Brown S (2016). The HubBle trial: Haemorrhoidal artery ligation (HAL) versus rubber band ligation (RBL) for symptomatic second- and third-degree haemorrhoids: a multicentre randomized controlled trial and health-economic evaluation. Health Technol Assess (Rockv).

[32] Shao WJ, Li GCH, Zhang ZHK, Yang BL, Sun GD, Chen YQ (2008). Systematic review and meta-analysis of randomized controlled trials comparing stapled haemorrhoidopexy with conventional haemorrhoidectomy. Br J Surg.

[33] MacRae HM, McLeod RS (1995). Comparison of hemorrhoidal treatment modalities - A meta-analysis. Dis Colon Rectum.

[34] Goligher JC (1980). ‘Surgery of the anus Rectum and colon. Bailliere Tindall.

[35] Gerjy R, Lindhoff-Larson A, Nyström PO (2008). Grade of prolapse and symptoms of haemorrhoids are poorly correlated: result of a classification algorithm in 270 patients. Color Dis.

[36] Rørvik HD (2019). Hemorrhoidal disease symptom score and short health scaleHD: New tools to evaluate symptoms and health-related quality of life in hemorrhoidal disease. Dis Colon Rectum.

[37] van Tol RR (2019). European Society of Coloproctology Core Outcome Set for haemorrhoidal disease: an international Delphi study among healthcare professionals. Color Dis.

